# Wearable-based assessment of anticipatory postural adjustments during step initiation in patients with knee osteoarthritis

**DOI:** 10.1371/journal.pone.0289588

**Published:** 2023-08-10

**Authors:** Luana Karine Resende Oliveira, Amélia Pasqual Marques, Yuzo Igarashi, Karen Flaviane Assis Andrade, Givago Silva Souza, Bianca Callegari

**Affiliations:** 1 Laboratório de Estudos da Motricidade Humana, Universidade Federal do Pará, Belém, PA, Brasil; 2 Associação das Pioneiras Sociais, Brasília, DF, Brasil; 3 Department of Physiotherapy, Speech Therapy and Occupational Therapy, Faculty of Medicine, University of São Paulo, São Paulo, Brazil; 4 Instituto de Ciências Biológicas, Universidade Federal do Pará, Belém, PA, Brazil; 5 Núcleo de Medicina Tropical, Universidade Federal do Pará, Belém, PA, Brasil; Universitatea de Medicina si Farmacie Victor Babes din Timisoara, ROMANIA

## Abstract

Older adults with moderate to severe knee osteoarthritis (KOA) exhibit adaptive strategy for initiating walking, known as anticipatory postural adjustments (APAs). While video motion kinematics has been the traditional way of measuring APAs, it can be difficult to transport and install, making it impractical for medical settings. Inertial sensors have become a more popular method for evaluating APAs, but no prior research has used accelerometers to measure gait initiation in individuals with KOA. The study aimed to assess the validity and reliability of a wearable accelerometer device for measuring APAs older adults with and without KOA. 25 individuals with KOA and 10 healthy individuals underwent evaluation using a wearable commercially available accelerometer (*MetamotionC)* and a video motion capture system. Reflective markers were placed on the lumbar vertebra and calcaneus. Participants were asked to initiate a step, and the researchers measured the APA_latency_ and APA_amplitude_ of each subject. APA_latency_ showed an very large to almost perfect correlation in both groups (CG:r = 0.82; p = 0.003 and KOA r = 0.98; p < 0.00001) between the instruments, while APA_amplitude_ had a moderate to very large correlation (CG: r = 0.65; p = 0.04and KOA: r = 0.80; p < 0.00001). Overall, the measurements showed fair to high reliability for intraclass correlation for video and accelerometer variables. Significant group effect was found for both variables: APA_latency_ (F_1, 66_ = 7.3; *p* = 0.008) and APA_amplitude_ (F_1,66_ = 9.5; *p* = 0.00). The wearable tri-axial accelerometer is a valid and reliable for assessing APAs during gait initiation in individuals with KOA, and this population exhibits lower APAs when initiating a step.

## Introduction

Gait initiation is a highly coordinated task that requires anticipatory postural adjustments (APAs) before the first step [1, 2]. APAs are required to start the step and occurs before the movement of wide displacement of the lower limbs (i.e., lifting the heel off the ground) to generate the forces and essential moments to drive the center of mass (COM) forward and toward the supporting limb. These APAs are generated by anticipated COM accelerations that lead to a posterior and lateral displacement of the center of pressure (COP) toward the heel of the swinging limb [2–4]. Individuals with knee osteoarthritis (KOA) have difficulty with weight-bearing tasks, including gait initiation [5, 6], which can result in reduced knee range of motion and speed [7].

To date, studies have shown that individuals with KOA have lower APA amplitude [2, 8, 9] and longer APA duration compared to healthy controls, indicating the adoption of different strategies for gait initiation [2], and that disease severity is related to APA parameter worsening [10]. However, these studies used cutting-edge technologies, such as video motion capture kinematics to measure COM and force platforms for measuring COP. Although they are considered the gold standard for obtaining reliable measurements, these technologies are expensive, difficult to transport, and require specialized management, which makes their use in clinical settings and sports centers impractical [11].

Accelerometers have been employed as a means to overcome this challenge and previous studies have investigated the detection of APAs using wearable sensors in different populations, such as subjects with Parkinson’s disease [4, 12] or healthy individuals [13, 14]. However, there is a need to validate the applicability of these methods specifically in the population with knee osteoarthritis (KOA). In the context of individuals with KOA, there are several factors that may affect the validity and reliability of previously developed methods for detecting APAs. Individuals with knee osteoarthritis (KOA) experience joint pain, stiffness, and reduced range of motion, leading to altered postural control strategies compared to healthy individuals [15] These changes can affect the timing, magnitude, and coordination of anticipatory postural adjustments (APAs) [9]. The severity of KOA, as indicated by Kellgren-Lawrence grades 2, 3, or 4, reflects varying degrees of structural damage and functional impairment, which can result in altered motor control strategies and compensatory movements [10]. The pathophysiological mechanisms of KOA, including joint inflammation, cartilage degeneration, and muscle weakness, can further contribute to biomechanical alterations during movement initiation [16]. These alterations have the potential to influence the initiation and execution of APAs in individuals with KOA. Consequently, it is necessary to validate APAs detection methods in this specific population, considering the unique characteristics and motor impairments associated with KOA. Validating these methods in individuals with KOA would provide valuable insights into the specific motor control alterations and potential therapeutic interventions for this patient group.

Thus, the present study sought to validate and test the reliability of a wearable commercial accelerometer to measure APAs during gait initiation in individuals with KOA. In addition, we aimed to compare APAs in older adults with and without KOA. We hypothesized that the accelerometer would be able to assess postural adjustments at gait initiation in these individuals, validating its usefulness relative to video motion capture system (the gold standard), with high reliability. We further hypothesized that older adults with KOA would present different APAs patterns, with lower APA amplitude and longer APA duration compared to the heathy subjects.

## Material and methods

### Subjects

This research study involved the participation of 25 individuals, both males and females, who were over the age of 50 and diagnosed with knee osteoarthritis (KOA). These individuals had Kellgren-Lawrence grades 2, 3, or 4 in at least one of their knees, indicating the severity of their condition [17]. The participants were referred to the study by orthopedists and/or rheumatologists from outside the research team. In addition to the KOA group, there were 10 healthy subjects who were carefully selected to match the characteristics of the KOA group. Subjects were divided accordingly into Control Group (CG) or Knee OA Group (KOA). Participants’ data are presented in Table 1.

**Table 1:** Clinical and demographic characteristics of the participants in both groups.

	KOA (n = 25)	CG (n = 10)
**Age, years**	58.80±5.08	55.50 ±5.44
**Height (m)**	1.60 ±0.05	1.56±0.07
**Weight**(**kg)**	75.13 ±13.89	70.30 ±12.41
**Sex, female/male**	17/8	6/4
**WOMAC, points**	39.55 ±19.28	-
**Laterality, number**		
**Bilateral**	18 (62.06%)	-
**Left**	7 (24.13%)	-
OA degree		
**2**	13 (44.82%)	-
**3**	12 (41.37%)	-
**4**	4 (13.79%)	-

BMI, body mass index; WOMAC, Western Ontario and McMaster Universities Osteoarthritis Index. Data are shown as mean (SD) or n (%).

The study performed in this investigation was approved by the Ethics Committee of the the Associação das Pioneiras Sociais (Report #4.698.811; CAEE: 44872721.5.0000.0022) as well as the Observational Studies in Epidemiology (STROBE) Statement. Written informed consent was obtained from all participants before the study was started, and all research was performed in accordance with Declaration of Helsinki. The subject screenings and evaluations were performed at the SARAH Belém Hospital (Belém, PA).

The inclusion criteria were: (1) knee OA with Kellgren–Lawrence grades 2, 3, or 4 in at least one knee, and (2) independent ambulation ability (3) right-footedness. Individuals with a previous history of orthopedic surgery, fractures, neurological diseases, self-reported cognitive difficulties affecting collection procedures, pregnancy, loss of protective foot sensation, and those who were not clinically stable were excluded from the study. Participants in the control group were right-footedness had no KOA and were recruited on a demand basis and selected for convenience, matched by demographic characteristics, and were excluded if they had any of the exclusion criteria above, or any other disease that could interfere with task performance.

### Instruments

Two instruments were used to measure and record the accelerations of the center of mass (COM) in the three axes during step initiation. A reflective marker was fixed on the fifth vertebra of the lumbar spine (L5) and on the calcaneus to mark the heel’s moment of departure from the ground at a sampling frequency of 120 Hz to record the COM accelerations in the axes of a three-dimensional system with three cameras (Simi Motion Capture; Simi, Germany). Video motion capture tracks the marker center coordinate and then the process involves calculating the first derivative of the marker position data twice, which provides an estimation of acceleration. A wireless inertial sensor positioned at L5 (*MetamotionC*; MbientLab) was also used. This triaxial device is approximately 25 mm × 4 mm in diameter, weighs only 0.2 g, has a 200 mAH replaceable battery, and involves the transfer of data via Bluetooth Low Energy Smart®. The acceleration range that the sensor captures is ±8g. COM acceleration data along the X-, Y-, and Z-axes were collected at 100 Hz.

### Experimental protocol

**Fig 1:**
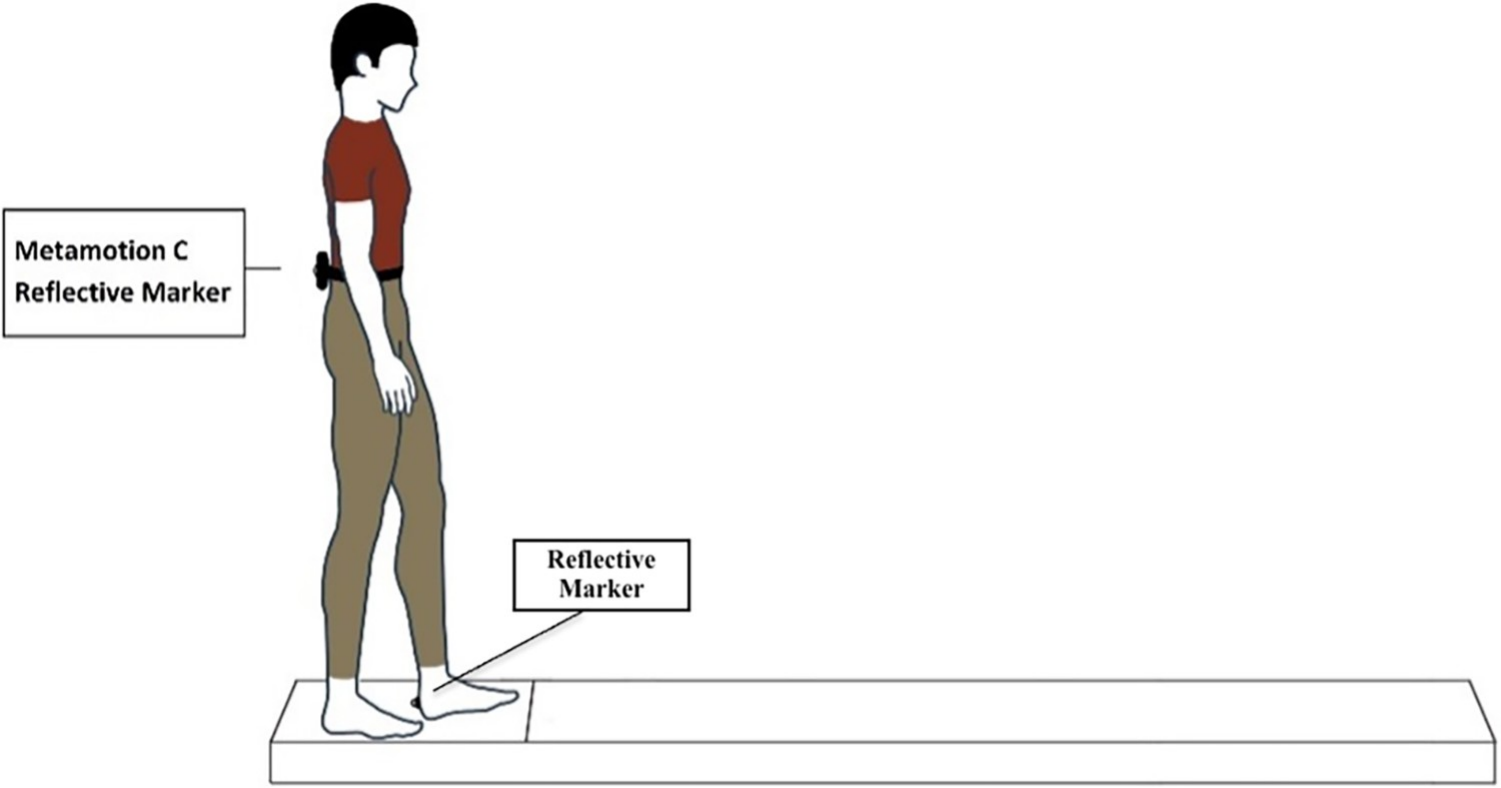
Standing individual with a wearable accelerometer attached at the L5 vertebra and the marker attached to the calcaneus and the L5 vertebra. The subject first stepped toward the 2-m walkway upon the researcher’s command.

The participants were instructed to stand barefoot in a standing position with their arms at their sides. The wearable accelerometer was fixed at L5 using a neoprene strap, and a reflective marker was placed on it (Fig 1). To synchronize the two assessment instruments, the participants were instructed to jump vertically in place prior to the start of the protocol to align the records performed by the peak of the acceleration signal on the vertical axis in both instruments, which characterizes the moment of impact with the ground.

The subjects remained standing on a 2-m walkway on marks drawn on the ground to control the initial foot position. The heels were mediolaterally separated by 6 cm and measured using a tape. The subject focused on an eye-level mark on the wall at a distance of 3 m and took a step forward with the right foot when he saw a light activated by the researcher. This was repeated ten times for each participant at random time intervals (0.5 – 4s) without prior announcements.

### Signal processing

**Fig 2:**
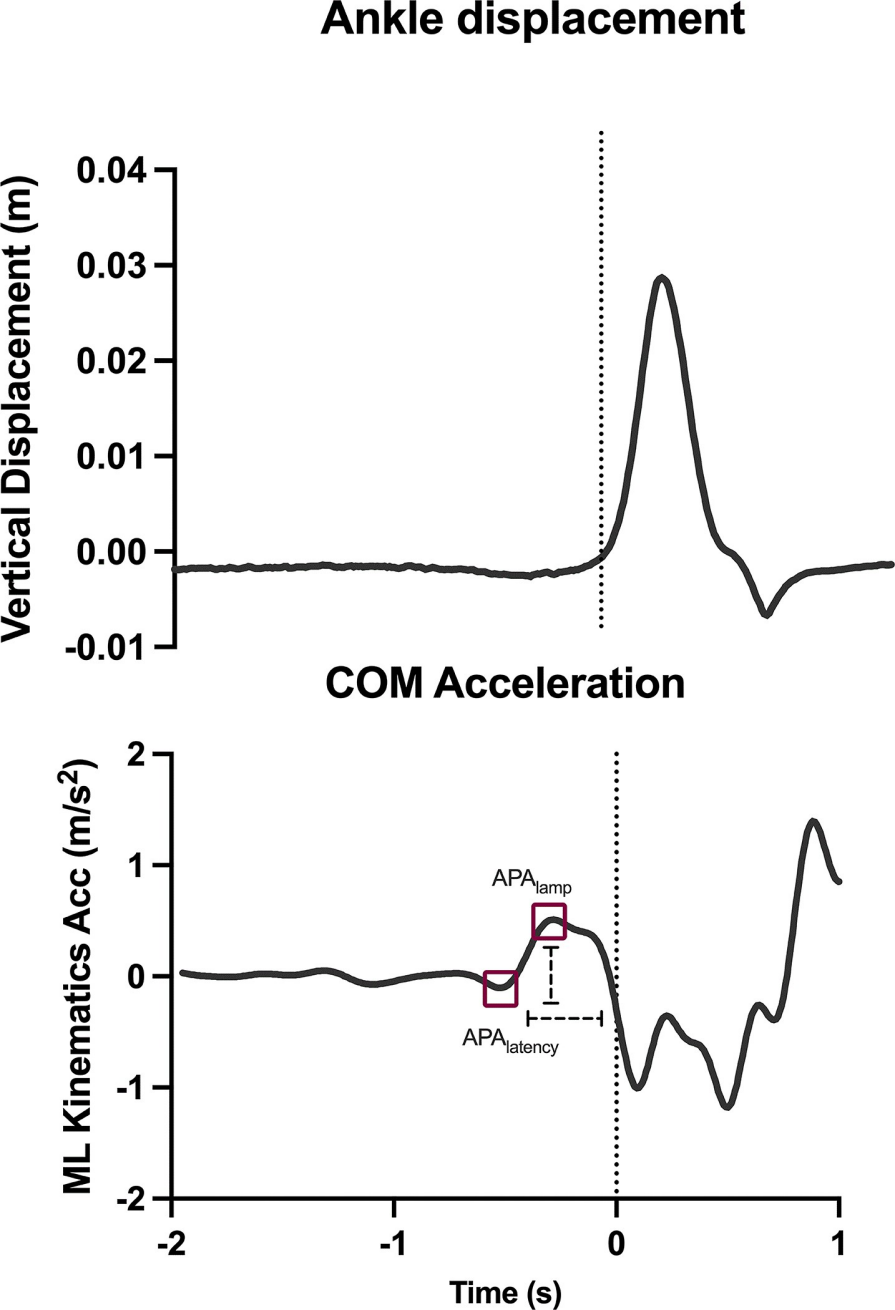
Upper panel: Ankle vertical displacement. Lower panel: Mediolateral (ML) acceleration curve extracted from the kinematic signal of one subject. The variables included in the method are expressed as red squares in the graph (explained in the Methods session). The dotted line represents the moment when the heel left the ground. COM: Center of Mass; APA_latency_: APA latency; APA_amp:_ APA amplitude.

The offline synchronization and analysis processes were performed using MatLab (MathWorks, Natick, MA, USA). The start of the step (Tzero) was defined as the moment when the heel left the ground. This was characterized by the instant when the vertical displacement of the reflective marker positioned at heel exceeded the mean of its baseline value (measured from the previous 500 ms) plus 2 standard deviations. With the demarcation of Tzero, the trials within each series were calculated for each participant. COM accelerations in the ML direction were analyzed and extracted from the records of the measuring instruments. The coordinates of the raw data on the ML axis were generated using video analysis and accelerometer. A 30 Hz low-pass second-order Butterworth filter was used to filter the signals from both instruments, and anticipatory parameters were calculated and exported (prior to Tzero) (Fig 2) as follows:

1. APA_latency_: APA latency, the moment at which the first mediolateral deviation occurs in acceleration data that exceeds two standard deviations above the baseline (measured from the previous 500 ms), before Tzero; and
2. APA_amplitude_ comprises the maximum mediolateral acceleration of the COM before the heel exit (Tzero).

### Statistical analysis

The mean and standard deviation of the APAonset (s) variable were calculated in a pilot trial involving 5 subjects from each group, resulting in values of -0.380s ± 0.04 for the control group and -0.470s ± 0.008 for the KOA group. To ensure a power test rate of 90% and an alpha level of 0.05, the sample size was determined to be 10 individuals in each group. Given the primary objective of validating the method specifically for knee osteoarthritis (KOA), the authors decided to increase the sample size of the KOA group. The statistical analysis was performed using GraphPad PRISM 9 software. The D’Agostino & Pearson test was used to analyze the normality of the variables. Data were normally distributed and described using a boxplot plot showing the median in the central line, the upper and lower edges (75th and 25th percentiles), and the minimum and maximum data values. The mean is plotted in the boxplot. A two-way ANOVA was performed to analyze the effect of the instrument (video motion kinematics or accelerometer) and group (Control or KOA) on APA_latency_ and APA_amplitude_. When significant effect was observed (i.e. group), follow-up t-tests were conducted running separate for each instrument (video motion kinematics or accelerometer), comparing each variable between groups.

For validation, the variables measured by each device were correlated using Pearson’s (r) correlation test. In the correlation tests, point-to-point agreement between systems was estimated per subject for each COM variable measured in each group, r values were estimated, and confidence intervals were reported (95%). Spearman’s correlation coefficients (r) were interpreted with magnitude thresholds of 0–0.1: trivial; 0.1–0.3: small; 0.3–0.5: moderate; 0.5–0.7: large; 0.7–0.9: very large; and 0.9–1.0: almost perfect [18]. Bland–Altman plots with 95% agreement limits (mean ± 2 SD) were plotted to compare the values of the equipment.

The reliability of intrasession recordings for both groups was calculated using the bidirectional random model of intraclass correlation coefficient (ICC), with a two-way random, single measures, absolute agreement and interpreted according to the classification of Shrout and Fleiss, where values of ICC ≥ 0.75 indicate excellent correlation, ICC = 0.74–0.4 indicate a fair to high correlation, and ICC ≤ 0.39 indicate low correlation [11]. Six trials were considered for the ICC calculation, since it was the minimum that all subjects achieved. The significance level was set at p < 0.05. The standard error of measurement (SEM) was calculated as the square root of the mean squared error term derived from the analysis of variance. The minimum detectable change (MDC) was considered the 95% CI of the SEM to estimate changes between each pair of measurements that could be clinically significant [19].

## Results

**Fig 3:**
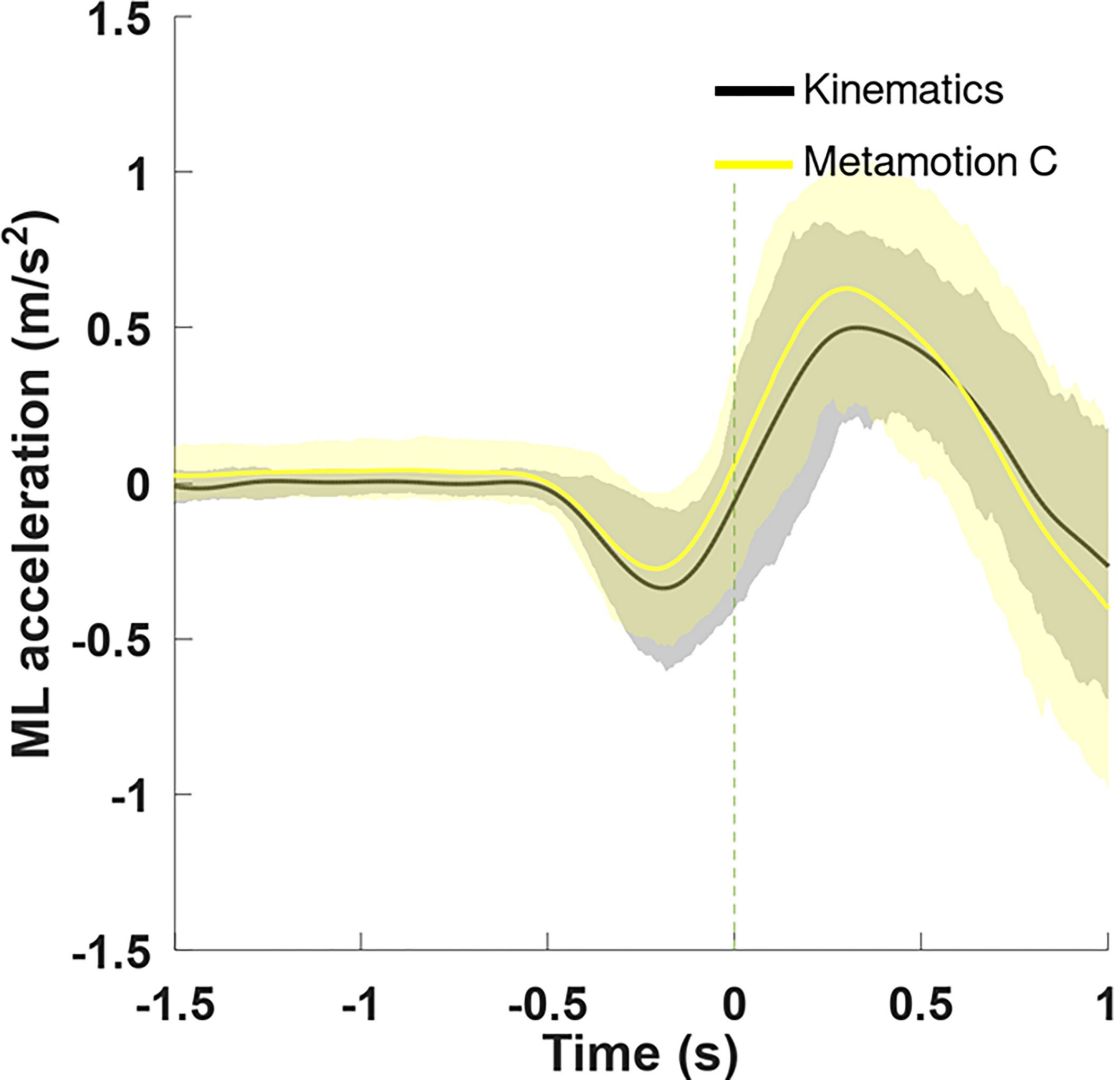
Center of mass accelerations from the six trials of all 35 subjects. Data from video kinematics and the accelerometer are represented. The thick line represents the average of all trials, while the dashed line represents the heel-off moment.

The similarity between both instruments is presented in Fig 3. Please note the average and standard deviation results for all subjects using both devices (accelerometer and video kinematics).

**Fig 4:**
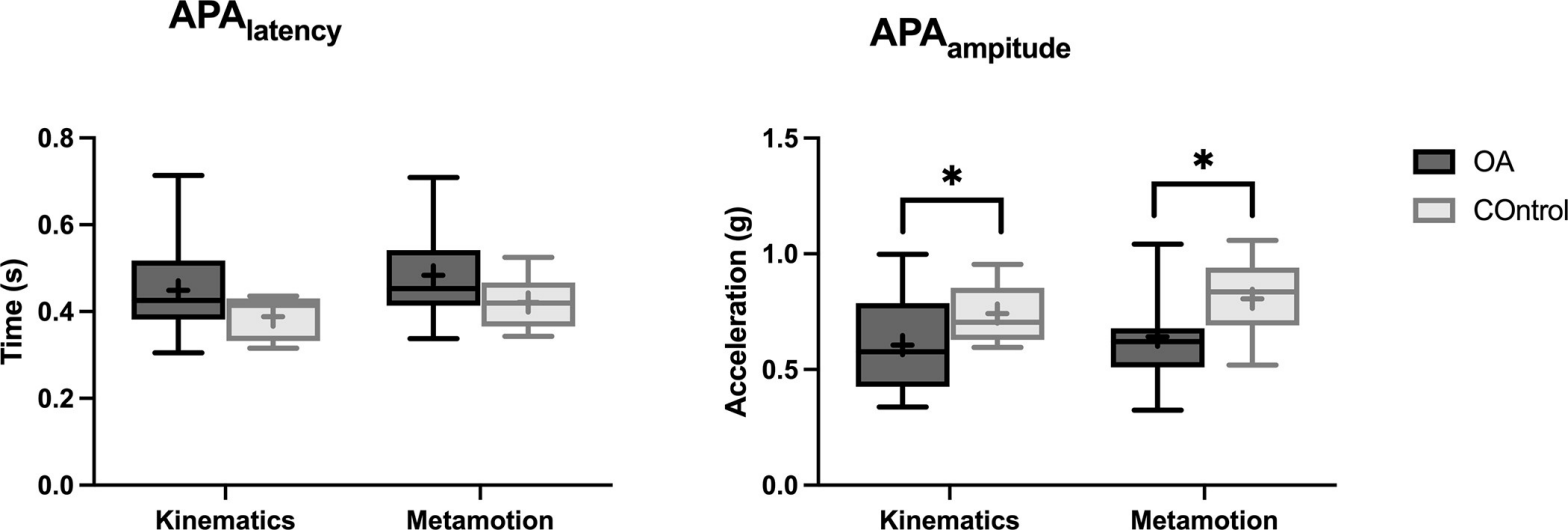
Analysis of the mean and standard deviation of the subjects in both groups, by instrument (video kinematics and the accelerometer). The study variables are represented above. A: APA_latency_; B: APA_amplitude_. APA, anticipatory postural adjustments.

Fig 4 shows the mean variables obtained from the records of both measurement instruments. No significant effect of the instrument (video kinematics and the accelerometer) was noted for APA_latency_ (F_(1,66_ = 2.2; *p* = 0.140) and APA_amplitude_ (F_(1,66)_ = 1.0; *p* = 0.309). However, group effect was significant for both variables: APA_latency_ (F _(1, 66_ = 7.3; *p* = 0.008) and APA_amplitude_ (F_(1, 66)_ = 9.5; *p* = 0.00). Interaction was not presented, either: APA_latency_ (F _(1, 66)_ = 0.00; *p* = 0.963) and APA_amplitude_ (F _(1, 66)_ = 0.0; *p* = 0.779).

Table two present mean (SD) values of the studied variables for both groups and instruments (Table 2). Between-group differences were found in APA_amplitude_ for both instruments, indicating that knee OA led to lower amplitudes in postural adjustments prior to step initiation.

**Table 2:** Mean (SD) values of the studied variables for both groups and instruments.

Variable	Instruments	KOA (Mean± SD)	CG (Mean± SD)	Mean Difference (95% CI)	*p-value*	*Eta squared*
**APA**_**latency (s)**_	video kinematics	0.45±0.10	0.39±0.05	-0.06 (-0.13 to 0.00)	0.10	0.10
	accelerometer	0.48 ± 0.10	0.42±0.06	-0.063 (-0.13 to 0.00)	0.06	0.10
**APA_amplitude (m/s^2^)_**	video kinematics	0.61 ± 0.19	0.74 ±0.12	0.14 (0.00 to 0.27)	0.04	0.12
	accelerometer	0.64 ± 0.20	0.81 ± 0.17	0.16 (0.02 to 0.31)	0.03	0.14

*Paired t-test for between-group comparison.

NOTE. Values are mean ± SD. Statistical comparisons were made with unpaired t test (The level of significance was p ≤ 0.05. Abbreviations: Control Group (CG) or Knee osteoarthritis Group (KOA)

### Validity

**Fig 5:**
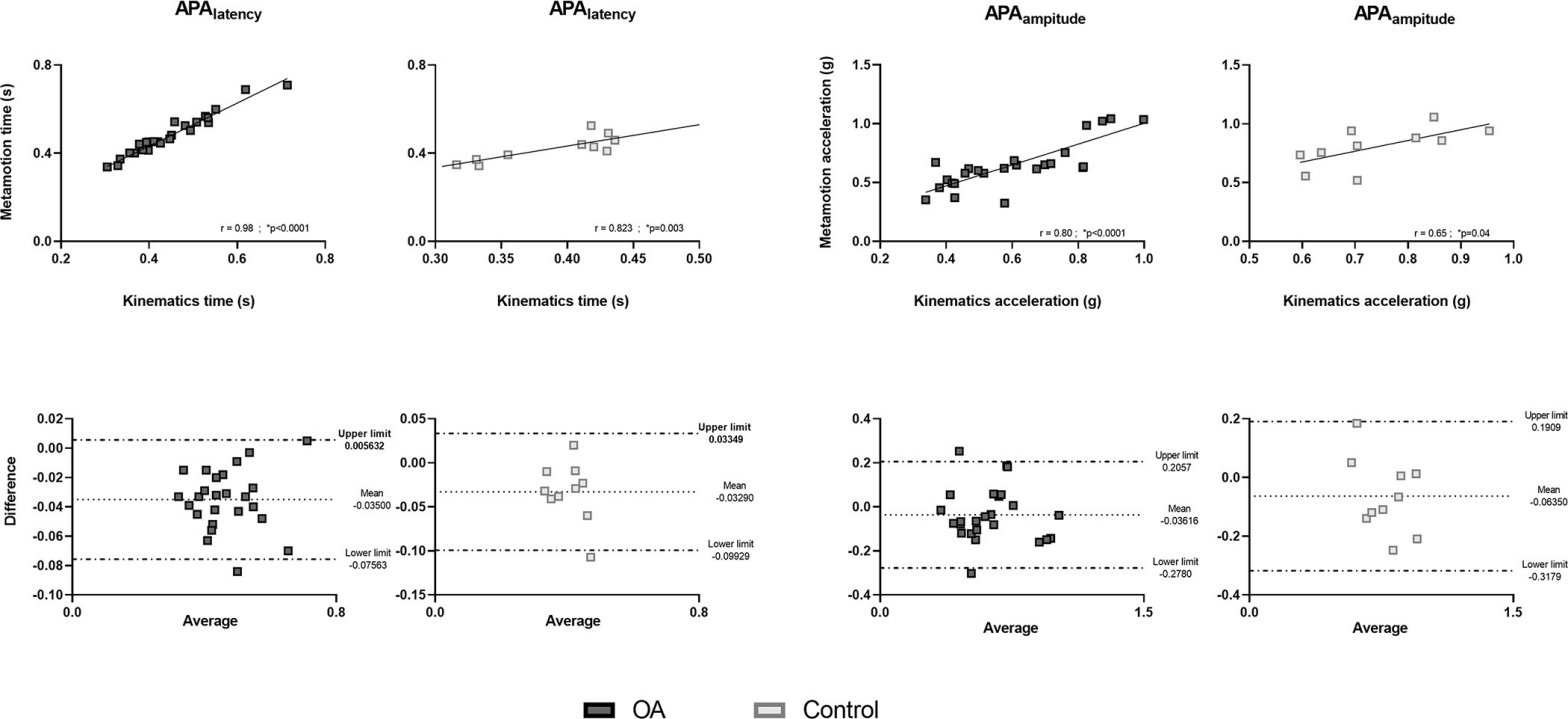
Linear correlation and Bland–Altman correlation graphs showing high correlation between the assessment instruments. A value of r ≥ 0.95 represents an almost perfect correlation, while a value of r ≥ 0.78 represents a very large correlation and the asterisk (*) represents the values that achieved statistical significance (p ≤ 0.001).

A linear correlation was observed between the measurements recorded using the two instruments. The variable APA_latency_ presented an almost perfect correlation (r = 0.98; p < 0.00001) in KOA group and very large correlation in control group (r = 0.82; p = 0.003), and the variable APA_amplitude_ had a very large correlation in KOA group (r = 0.80; p < 0.00001) and moderate correlation in control group (r = 0.65; p = 0.04). The agreement between the measures can also be verified in the Bland–Altman graphs because the average of the differences presents little dispersion. The results, therefore, indicate that the accelerometer was able to assess postural adjustments at gait initiation (Fig 5).

### Reliability

The measurements found in this study showed fair to high reliability [20] in the ICC for the video motion kinematics and accelerometer APA_latency_ and APA_amplitude_ variables (Table 3). It is also possible to notice similarities in the SEM and MDC between the measurement instruments, demonstrating comparable sensitivities.

**Table 3:** Intra-session reliability of the video kinematic versus accelerometer assessment methods.

Intra-session reliability								
	ICC		Confidence Intervals		SEM		MDC	
	KOA	CG	KOA	CG	KOA	CG	KOA	CG
**APAamplitude(s)**								
Video kinematic	0.552*	0.549*	0.383; 0.729	0.291; 0.824	0.035	0.036	0.080	0.084
Accelerometer	0.561*	0.613*	0.393; 0.735	0.364; 0.855	0.004	0.005	0.009	0.011
**APAlatency (m/s** ^**2**^ **)**								
Video kinematic	0.526*	0.192*	0.356; 0.710	0.009; 0.549	0.019	0.030	0.044	0.069
Accelerometer	0.470*	0.381*	0.299; 0.664	0.145; 0.715	0.019	0.023	0.043	0.053

NOTE. APA, anticipatory postural adjustments; ICC, intraclass correlation coefficient; MDC, minimum detectable change; SEM: standard error of measurement. *p < 0.001.

## Discussion

The present study aimed to evaluate the validity and reliability of the wearable commercial accelerometer versus video motion kinematics for measuring APAs during gait initiation in older adults with and without KOA. We hypothesized that the commercially available accelerometer can evaluate anticipatory postural adjustments at gait initiation in these individuals, with good reliability and validation relative to the gold standard. We further hypothesized that older adults with KOA would present different APAs patterns, with lower APA amplitude and longer APA duration compared to the heathy subjects. The results confirmed our initial hypothesis because APA_latency_ showed avery large to almost perfect correlation, while APA_amplitude_ had a moderate to very large correlation between the measurements of both instruments. Therefore, the results indicate that the accelerometer was able to assess postural adjustments at gait initiation. The measurements also showed fair to high reliability for ICC for the kinematic and accelerometer amplitude and latency variables. Concerning the comparison between groups (GC and KOA), our hypothesis was partially confirmed since there was a significant effect of group in both variables assessed, although follow up comparison only demonstrates lower APA amplitude in KOA group compared to control.

Although several studies have validated the use of inertial sensors for analyzing the spatial-temporal aspects of gait in patients with knee osteoarthritis (KOA) [15, 21], comparing the results of our study with the existing literature has been challenging due to the lack of previous studies utilizing inertial sensors to assess anticipatory postural adjustments during gait initiation in KOA patients. However, the importance of using accelerometers to evaluate gait initiation in other populations is evident from multiple studies. For instance, Martinez-Mendez et al. (2011) employed inertial wearable sensors to detect anticipatory postural adjustments preceding gait initiation, providing valuable insights into motor control mechanisms [4]. Bonora et al. (2017) utilized wearable inertial sensors to analyze anticipatory postural adjustments in individuals with Parkinson’s disease, revealing impairments during gait initiation [22]. Furthermore, da Costa Moraes et al. (2022) validated a smartphone app for assessing postural adjustments during step initiation, highlighting the potential widespread use of accelerometer-based assessments [14]. Additionally, Gazit et al. (2020) introduced a novel approach using wearable sensors to quantify gait initiation, contributing to a deeper understanding of pre-movement dynamics [13]. Taken together, these studies emphasize the significance of accelerometer-based assessments in evaluating gait initiation in KOA patients, providing valuable insights into motor control and potential clinical applications.

From the moment gait initiation occurs, denoted by the shift from a static to dynamic state, the systems accountable for postural control face considerable challenges [23], APAs play a crucial role in averting substantial destabilization [23, 24]. Consequently, the evaluation of knee osteoarthritis (KOA) population benefits significantly from considering this indicator. Our findings substantiate those individuals with knee osteoarthritis exhibited diminished amplitudes in APAs. The occurrence of falls in older individuals during short-distance travel is indicative of challenges in maintaining stability during this transitional phase [25]. Older individuals affected by neurological or musculoskeletal conditions, such as osteoarthritis (OA), may exhibit alterations in balance control that contribute to gait difficulties. Consequently, limitations in both gait and postural balance serve as significant predisposing factors for an elevated risk of falls among the older adults [23, 26, 27].

Video motion kinematics is considered the gold standard for obtaining reliable postural control measurements; however, it is expensive, difficult to transport, and requires proper camera installation and calibration, making its use impractical in clinics and sports centers. When assessing Anticipatory Postural Adjustments (APAs) during step initiation, it is indeed common to use the acceleration of the center of mass (COM) [13, 14], thus, the present study validated the use of inertial sensors to assess APAs in patients with KOA and showed high reliability, demonstrating that they can be an alternative assessment, facilitating its implementation.

The present study had some limitations. Uncontrolled spatiotemporal gait parameters such as velocity, cadence, and stride variability may have impacted the validity of the study’s outcomes. Moreover, the absence of an internationally recognized protocol for gait initiation may have hindered the generalization of the study’s hypothesis. Nonetheless, this research contributes to the existing literature by presenting novel data on the utilization of accelerometers for assessing Anticipatory Postural Adjustments (APAs) in individuals with knee osteoarthritis (KOA), as well as comparing the signals obtained from various measuring devices.

This study confirmed the validity and reliability of the wearable accelerometer versus video motion kinematics for gait initiation in individuals with KOA. This population also presented lower amplitudes in postural adjustments to initiate a step. Thus, there is applicability of this device in clinical environments since it is an instrument of lower cost and easy instrumentation.

## Supporting information

S1 Data (XLSX)
